# SHIP-Deficient Dendritic Cells, Unlike Wild Type Dendritic Cells, Suppress T Cell Proliferation via a Nitric Oxide-Independent Mechanism

**DOI:** 10.1371/journal.pone.0021893

**Published:** 2011-07-06

**Authors:** Frann Antignano, Melisa Hamilton, Scott Patterson, Victor Ho, Carla Cohen, Megan K. Levings, Gerald Krystal

**Affiliations:** 1 The Terry Fox Laboratory, British Columbia Cancer Agency, Vancouver, British Columbia, Canada; 2 The Biomedical Research Centre, The University of British Columbia, Vancouver, British Columbia, Canada; 3 Department of Surgery, The University of British Columbia and Immunity in Health and Disease, Child and Family Research Institute, B.C. Children's Hospital, Vancouver, British Columbia, Canada; McGill University, Canada

## Abstract

**Background:**

Dendritic cells (DCs) not only play a crucial role in activating immune cells but also suppressing them. We recently investigated SHIP's role in murine DCs in terms of immune cell activation and found that TLR agonist-stimulated SHIP−/− GM-CSF-derived DCs (GM-DCs) were far less capable than wild type (WT, SHIP+/+) GM-DCs at activating T cell proliferation. This was most likely because SHIP−/− GM-DCs could not up-regulate MHCII and/or co-stimulatory receptors following TLR stimulation. However, the role of SHIP in DC-induced T cell suppression was not investigated.

**Methodology/Principal Findings:**

In this study we examined SHIP's role in DC-induced T cell suppression by co-culturing WT and SHIP−/− murine DCs, derived under different conditions or isolated from spleens, with αCD3+ αCD28 activated WT T cells and determined the relative suppressive abilities of the different DC subsets. We found that, in contrast to SHIP+/+ and −/− splenic or Flt3L-derived DCs, which do not suppress T cell proliferation *in vitro*, both SHIP+/+ and −/− GM-DCs were capable of potently suppressing T cell proliferation. However, WT GM-DC suppression appeared to be mediated, at least in part, by nitric oxide (NO) production while SHIP−/− GM-DCs expressed high levels of arginase 1 and did not produce NO. Following exhaustive studies to ascertain the mechanism of SHIP−/− DC-mediated suppression, we could conclude that cell-cell contact was required and the mechanism may be related to their relative immaturity, compared to SHIP+/+ GM-DCs.

**Conclusions:**

These findings suggest that although both SHIP+/+ and −/− GM-DCs suppress T cell proliferation, the mechanism(s) employed are different. WT GM-DCs suppress, at least in part, via IFNγ-induced NO production while SHIP−/− GM-DCs do not produce NO and suppression can only be alleviated when contact is prevented.

## Introduction

Dendritic cells (DCs) have long been recognized as activators of the immune system [1]–[3] and, more recently, as critical players in the induction of central tolerance [4] as well as the induction and maintenance of peripheral tolerance [5]. Several signals are involved in determining the nature of the interaction between T cells and DCs, including the antigen (Ag)-specific interaction between the T cell receptor (TCR) on T cells and the peptide-bound major histocompatibility complex molecule on DCs, contact mediated signals transduced by co-stimulatory or tolerogenic receptors and secreted cytokines [6]. Under normal steady state conditions, DCs maintain tolerance by either inducing Tregs [7] or by causing deletion or anergy of self-reactive T cells [8]. DCs with these suppressive properties can be generated *in vitro* and have many potential applications, such as in the treatment of autoimmune disorders or organ transplants. A greater understanding of the mechanisms involved will allow tailoring of DCs for specific applications.

Currently very little is known about the mechanisms that DCs employ to suppress T cell proliferation. In one report, DCs derived from rat bone marrow (BM) using granulocyte macrophage colony stimulating factor (GM-CSF) ± interleukin (IL)-4 were shown to have an intrinsic ability to prevent T cell proliferation while those derived using fms-like tyrosine kinase ligand (Flt3L) did not [9]. However, the specific mechanism of DC-mediated suppression was not determined in this study. In another study, using myeloid dendritic cell (mDC) precursors, isolated as CD11c^−^ cells from GM-CSF cultures, these cells were shown to suppress T cell proliferation via a contact-dependent, NO-mediated mechanism [10]. In addition, DCs that were exposed to tumor cells were found to become immunosuppressive by down-regulating the TCR component CD3ε on T cells, and by inducing reactive oxygen species (ROS)-mediated T cell apoptosis [11]. Taken together, these data illustrate a diversity of mechanisms available to DCs to induce T cell suppression. Importantly, the environment in which the DCs are generated appears to influence both their ability to suppress and the suppressive mechanism they employ.

Interestingly, immune suppression is not a function associated only with DCs. Several cell types including regulatory T cells (Tregs) [12] and tumor-induced myeloid-derived suppressor cells (MDSCs), which are characterized by the co-expression of Gr1 and CD11b [13], are capable of immune suppression. The mechanisms of suppression used by these cells are quite diverse. Suppression by Tregs, for example, is often associated with either membrane bound- or secreted TGFβ-induced anergy [14], cytokine deprivation-mediated apoptosis [15] and/or contact-dependent cell death, involving granzyme B [16]. MDSCs, on the other hand, often use a different arsenal of suppressive mechanisms, including arginase 1 (Arg 1) [17]. Related to this, the amino acid, L-arginine, can be metabolized by inducible nitric oxide synthase (iNOS, also known as NOS2), into nitric oxide (NO), or it can be converted into L-ornithine by the enzyme Arg 1 [18]. Co-expression of these two enzymes can lead to the generation of reactive nitrogen-oxide species (RNOS) such as peroxynitrite which, in turn, nitrosylates the TCR and other proteins, causing T cell suppression [19].

The SH2-containing inositol 5′ phosphatase (SHIP) is a critical negative regulator of the phosphoinositol 3-kinase (PI3K) pathway with known functions in regulating myeloid cell development and survival [20]. Recently, we showed that SHIP-deficient DCs, generated in the presence of GM-CSF, were less mature than wild type (WT, SHIP+/+) DCs and were far less able to up-regulate MHCII and co-stimulatory receptors in response to Toll like receptor (TLR) activation than WT GM-CSF derived DCs (GM-DCs) and this resulted in these SHIP−/− GM-DCs being far less able to induce Ag-specific T cell proliferation [21]. However, we did not look at SHIP's role in the suppressive ability of various DC subsets. In this study, we were interested in determining if the inability of SHIP−/− DCs to activate T cells translated into an enhanced suppressive ability. Specifically, we compared the ability of SHIP +/+ and −/− DCs derived with GM-CSF, Flt3L (FL-DCs) or isolated from spleens to suppress polyclonally activated T cells in order to ascertain the role of SHIP in DC-induced T cell suppression. Our results reveal that naïve WT and SHIP-deficient GM-DCs suppress T cell proliferation to the same extent while SHIP+/+ and −/− Flt3L-derived or splenic DCs do not suppress at all. Moreover, we discovered that SHIP−/− GM-DCs express Arg 1 and do not produce NO, while WT GM-DCs do not express Arg 1 and suppress T cell proliferation, in part, via NO-production.

## Results

### WT and SHIP−/− GM-CSF-derived DCs are equally suppressive

**Figure 1:**
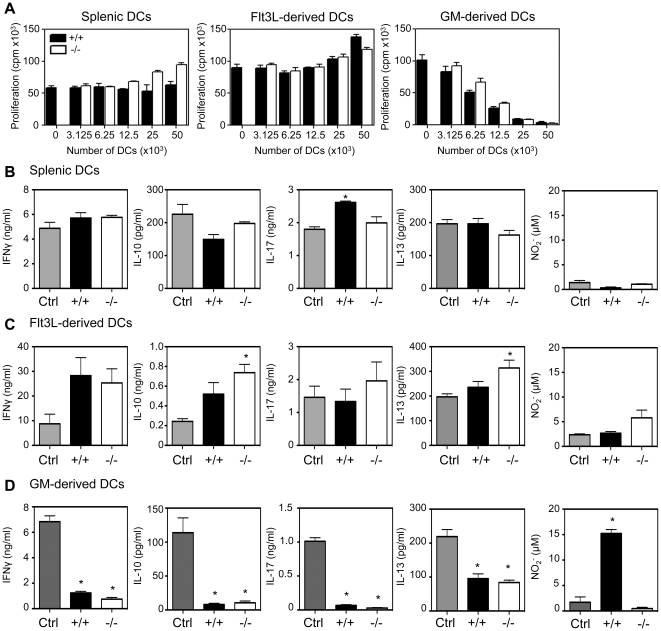
SHIP+/+ and −/− GM-DCs suppress T cell activation. 2×10^5****^WT splenocytes were stimulated with soluble αCD3+ αCD28 antibodies and incubated with the indicated number of SHIP+/+ or −/− **A)** CD11c^+^ splenic DCs, FL- or GM-DCs. Proliferation was determined after 72 hrs by incorporation of ^3^H-thymidine for the last 18 hrs. Data shown are the mean ± SEM of triplicate cultures and are representative of more than 3 independent experiments. Supernatants were collected after 72 hrs from **B)** Splenic (25×10^3^) **C)** Flt3L-derived (25×10^3^) and **D)** GM-derived DCs (50×10^3^) co-cultures and subjected to cytokine ELISAs or Griess assays for NO determination. Data shown are the mean ± SEM of triplicate cultures and are representative of 2–3 independent experiments. *p<0.05 relative to stimulated splenocytes in the absence of DCs (Ctrl).

To test whether WT or SHIP−/− DCs isolated from the spleen or derived under different culture conditions had suppressive activity, we cultured different cell concentrations of these DCs with splenic T cells activated with αCD3+ αCD28. As shown in Fig 1A, SHIP+/+ and −/− DCs isolated from the spleen or derived using Flt3L did not suppress T cell proliferation at any DC dose tested. In contrast, SHIP+/+ and −/− GM-DCs suppressed T cell proliferation in a similar, cell dose dependent manner, with greater than 50% suppression achieved with the addition of 12.5×10^3^ DCs to 2×10^5^ WT splenocytes (Fig 1A). Unlike our previous study in which we found that SHIP−/− GM-DCs were far less capable than WT GM-DCs at activating T cell proliferation [21], these results show that SHIP+/+ and −/− GM-DCs are equally potent at suppressing T cell proliferation.

In addition to T cell proliferation, we also analyzed cytokine secretion from αCD3+ αCD28 stimulated WT spleen cells co-cultured with SHIP+/+ or −/− DCs and found that the production of the T cell cytokines IFNγ, IL-10, IL-17 and IL-13 correlated with our T cell proliferation results, ie, when activated T cells were co-cultured with either splenic DCs or FL-DCs there was no reduction in IFNγ, IL-10, IL-17 or IL-13 (Fig 1B and C) but when αCD3+ αCD28 stimulated WT spleen cells were co-cultured with SHIP+/+ or −/− GM-DCs, the levels of these cytokines were significantly reduced (Fig 1D). In addition to cytokines, we also determined the NO levels produced in the co-cultures and found the addition of SHIP+/+ GM-DCs significantly increased NO levels. Interestingly, however, addition of SHIP−/− GM-DCs resulted in very little NO production (Fig 1D, far right panel). Importantly, when activated T cells were co-cultured with either splenic DCs or FL-DCs there was very little NO secreted (Fig 1B and C, far right panel).

### WT GM-DCs suppress, in part, by an NO-dependent mechanism

**Figure 2:**
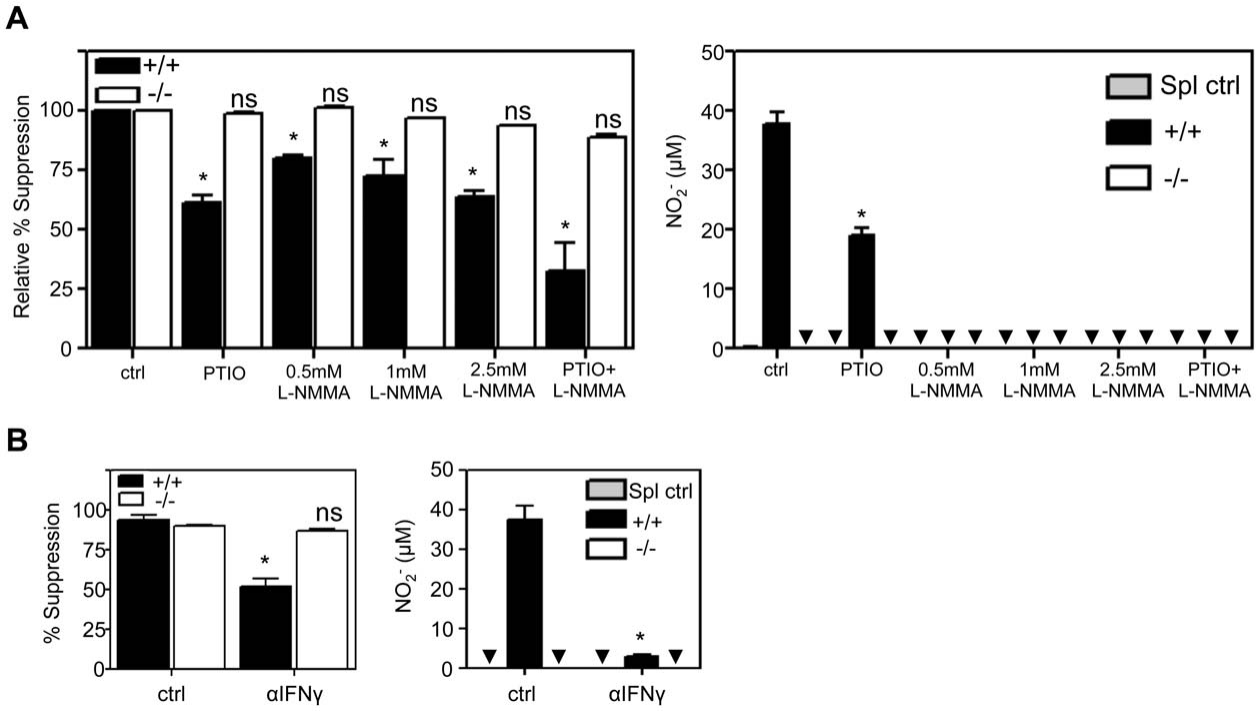
SHIP+/+ but not −/− GM- DC-induced T cell suppression is mediated by IFNγ-dependent NO production. WT splenocytes were stimulated with soluble αCD3+ αCD28 Abs and incubated with SHIP+/+ or −/− GM-DCs (50×10^3^) for 72 hrs. **A)** Left panel, relative percent suppression of proliferation in the absence (ctrl) or presence of 25 µg/ml PTIO, 0.5 mM–2.5 mM L-NMMA or 25 µg/ml PTIO +0.5 mM L-NMMA). Right panel, NO production using the same concentrations of PTIO and/or L-NMMA. **B)** WT splenocytes were stimulated with soluble αCD3+ αCD28 Abs and incubated with SHIP+/+ or −/− GM-DCs (50×10^3^) ±10 µg/ml neutralizing αIFNγ. Left panel, percent suppression of T cell proliferation. Right panel, NO production. Data shown are mean ± SEM of triplicate cultures and are representative of 3 independent experiments. *p<0.05 relative to genotype control, ns  =  not significantly different. ▾ indicates level is below detection.

Since WT CD11c^−^ mDC precursors have been shown to prevent T cell proliferation via an NO-mediated mechanism [10], we investigated whether NO was involved in the suppression mediated by SHIP+/+ or −/− GM-DCs. Specifically, we asked if an NO scavenger, carboxy PTIO, or an iNOS inhibitor, L-NMMA, could ameliorate the T cell suppression induced by these GM-DCs. As can be seen in Fig 2A (left panel), the addition of carboxy PTIO significantly reduced the level of WT GM-DC-induced suppression, as did the addition of L-NMMA in a dose dependent manner. A combination of the two caused a dramatic reduction. These inhibitors, however, had no effect on the ability of SHIP−/− GM-DCs to suppress WT T cell proliferation (Fig 2A), consistent with their inability to produce NO in co-cultures (Fig 1B). The reduction in WT GM-DC-induced suppression via carboxy PTIO also correlated with a reduction in NO production while L-NMMA prevented any detectable levels of NO at all doses tested (Fig 2A, right panel). Worthy of note, however, is that while L-NMMA, even at 0.5 mM, completely eliminated NO secretion it only reduced T cell suppression by approximately 25%, suggesting that a mechanism of suppression other than NO was also being used by WT GM-DCs. Since NO-dependent mechanisms employed to suppress T cell proliferation have been reported to often involve IFNγ, which is secreted by activated T cells and induces iNOS expression in macrophages and DCs [10], [22]–[25] we tested the effect of a neutralizing Ab to IFNγ and found that SHIP+/+ GM-DCs were significantly less capable of suppressing T cell proliferation in the presence of this Ab and that less NO was generated in these co-cultures (Fig 2B). No effect was observed in SHIP−/− cultures, consistent with the inability of SHIP−/− DCs to induce NO.

### SHIP−/− GM-DCs express Arg 1 but suppression is not mediated by amino acid depletion

**Figure 3:**
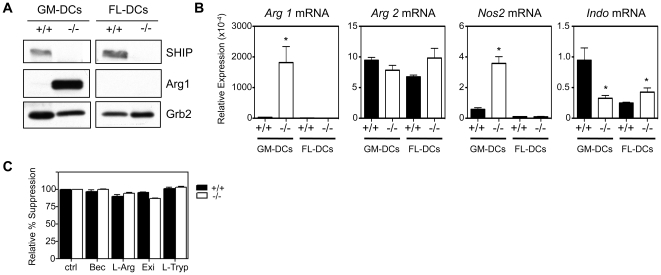
SHIP−/− GM-DCs express Arg 1. **A)F** Day 8 SHIP+/+ and −/− GM- and FL-DCs were subjected to Western analysis using Abs to SHIP, Arg1 and Grb2 as a loading control. Data shown are representative of at least 3 independent experiments. **B)** mRNA expression of *Arg 1*, *Arg 2*, *Nos2*, and *Indo* in SHIP+/+ and SHIP−/− GM- and FL-DCs. Data shown is mean ± SEM of duplicate determinations from 2–3 independent experiments. *p<0.05 relative to SHIP+/+. **C)** WT splenocytes were stimulated with soluble αCD3+ αCD28 Abs and incubated with SHIP+/+ or −/− GM-DCs (50×10^3^) ±100 µM of the arginase inhibitor, Bec, 2 mM L-arginine (L-Arg), 1 µM of the IDO inhibitor, exiguamine A (Exi) or 200 µM L-tryptophan (L-Tryp). Data shown are the mean ± SEM of triplicate determinations and are representative of 2 independent experiments.

The expression of Arg 1 in macrophages and MDSCs has been shown to be one mechanism by which these cells suppress T cell proliferation and this enzyme suppresses via sequestering L-arginine away from iNOS and converting it into L-ornithine instead of NO [13], [17], [18]. Related to this, SHIP−/− peritoneal macrophages have been shown to have an alternatively activated, immunosuppressive M2 phenotype, characterized by high Arg 1 expression and this has been linked to enhanced tumor growth in SHIP-deficient mice [26]. In addition, we have found that GM-CSF- and IL-3-derived SHIP−/− macrophages express high levels of Arg1 as a result of basophil produced IL-4 [27]. We therefore examined the expression of Arg 1 in SHIP+/+ and −/− DCs derived under different culture conditions (Fig 3A). We found that SHIP−/− DCs derived in the presence of GM-CSF expressed Arg 1, likely as a result of SHIP−/− basophil produced IL-4 [27], while WT GM-DCs did not. In contrast, neither SHIP+/+ nor −/− DCs expressed Arg 1 when derived with Flt3L (Fig 3A). We also looked at *Arg 1*, *Arg 2* and *Nos2* (iNOS) mRNA levels in naive SHIP+/+ and −/− GM-DCs and FL-DCs by qPCR. We found increased expression of *Arg 1* and *iNOS* in SHIP−/− GM-DCs compared to SHIP+/+ GM-DCs while both SHIP+/+ and −/− FL-DCs expressed very low levels of *Arg 1* and *iNOS* (Fig 3B). While it is interesting that SHIP−/− GM-DCs express higher iNOS levels than WT GM-DCs, at least at the mRNA level in naive DCs, the fact that SHIP−/− GM-DCs also express very high levels of Arg 1 likely prevents them from producing significant amounts of NO. Also of interest, no significant differences were detected in mRNA levels of *Arg 2* between SHIP+/+ or −/− GM or FL-DCs (Fig 3B).

Since suppression of T cell proliferation can be achieved through the expression of Arg 1 and subsequent depletion of L-arginine [28] we asked if SHIP−/− GM-DCs were suppressing T cell proliferation via this mechanism by using the Arg 1 inhibitor, BEC ([S]-[2-boronoethyl]-L-cysteine-HCl). However, we found that there was no reversal of suppression in either the SHIP+/+ or −/− DC co-cultures (Fig 3C). We also added exogenous L-arginine to cultures and found no reversal of suppression. The amino acid tryptophan, which is the rarest essential amino acid and thus may cause a “bottle-neck” in protein synthesis, has also been reported, upon local depletion, to cause T cell anergy and death [29], [30]. Related to this, indoleamine 2,3-dioxygenase (IDO, or *Indo*), a key tryptophan-degrading enzyme, is expressed by some DC subsets [29] and generates tryptophan catabolites that can lead to T cell apoptosis [31]. We therefore determined the expression of *Indo*, and found that it was expressed at very low levels in SHIP+/+ and −/− GM-DCs and FL-DCs, with SHIP+/+ GM-DCs having the highest expression (Fig 3B, far right panel). To test if IDO played a role in either SHIP+/+ or −/− GM-DC-induced T cell proliferation we added an IDO inhibitor, exiguamine A [32], to co-cultures but found that inhibiting IDO had no effect, nor did the addition of exogenous L-tryptophan (Fig 3C).

### Neither SHIP+/+ nor −/− GM-DCs suppress T cell proliferation via secreted immunosuppressive cytokines

**Figure 4:**
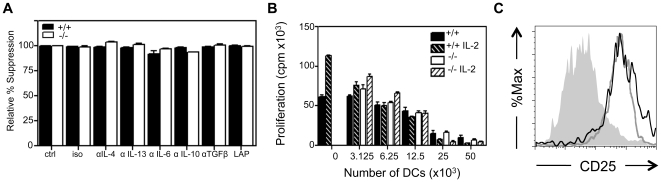
Secreted cytokines are not responsible for T cell suppression. **A)** WT splenocytes (2×10^5^) were stimulated with soluble αCD3+ αCD28 Abs and co-incubated with SHIP+/+ or −/− GM-DCs (50×10^3^) containing isotype control Ab (iso) or the indicated neutralizing cytokine Ab (10 µg/ml) or LAP (250 ng/ml). **B)** WT splenocytes were stimulated with soluble αCD3+ αCD28 Abs and incubated with the indicated number of SHIP+/+ and −/− GM-DCs and IL-2 (100 U) was added as indicated and proliferation determined after 72 hrs. Data shown are the mean ± SEM of triplicate cultures and is representative of at least 2 independent experiments. **C)** CD4^+^ T cells from SHIP+/+ and −/− GM-DC (50×10^3^) co-cultures were analyzed for expression of CD25 by flow cytometry. Splenocyte control  =  grey fill, WT GM-DCs  =  black line and SHIP−/− GM-DCs  =  grey line. Data shown are representative of 2 independent experiments.

Apart from depleting amino acids in the local milieu, T cell suppression is often mediated by local secretion of immunosuppressive cytokines. Therefore, we neutralized several cytokines with known or potential suppressive functions. However, addition of neutralizing antibodies to IL-4, IL-13, IL-6, IL-10 and TGFβ, resulted in no amelioration of T cell suppression induced by either SHIP+/+ or −/− GM-DCs (Fig 4A). As well, since TGFβ is often membrane bound and expressed at the cell surface in a latent form via its non-covalent association with latency associated peptide (LAP) [33], we added exogenous LAP to retain TGFβ in an inactive state. This too had no effect on the level of T cell suppression (Fig 4A). These results suggest that the DC secreted cytokines, IL-4, IL-13, IL-6, IL-10 and TGFβ, or membrane-bound TGFβ, are not responsible for the T cell suppression induced by SHIP+/+ or −/− GM-DCs.

IL-2 is an important autocrine-acting cytokine that T cells produce to promote their own proliferation [34]. We therefore asked if the suppression of T cell proliferation induced by SHIP+/+ or SHIP−/− GM-DCs was occurring via inhibition of IL-2 production. To test this, we added exogenous IL-2 to co-cultures of αCD3+ αCD28 stimulated WT splenocytes with SHIP+/+ or−/− GM-DCs and found that this enhanced T cell proliferation in the absence of DCs, but did not abrogate suppression when DCs were present (Fig 4B). We also tested whether SHIP+/+ or −/− GM-DCs were reducing IL-2R expression on the T cells. However, the expression of CD25 (the IL-2Rα) on CD4^+^ T cells was increased compared to controls when either SHIP+/+ or −/− GM-DCs were present (Fig 4C). Therefore, neither SHIP+/+ nor −/− GM-DCs were reducing the ability of WT splenic T cells to use IL-2 via down-regulation of its receptor.

### SHIP+/+ and −/− GM-DCs suppress via a contact-dependent mechanism

**Figure 5:**
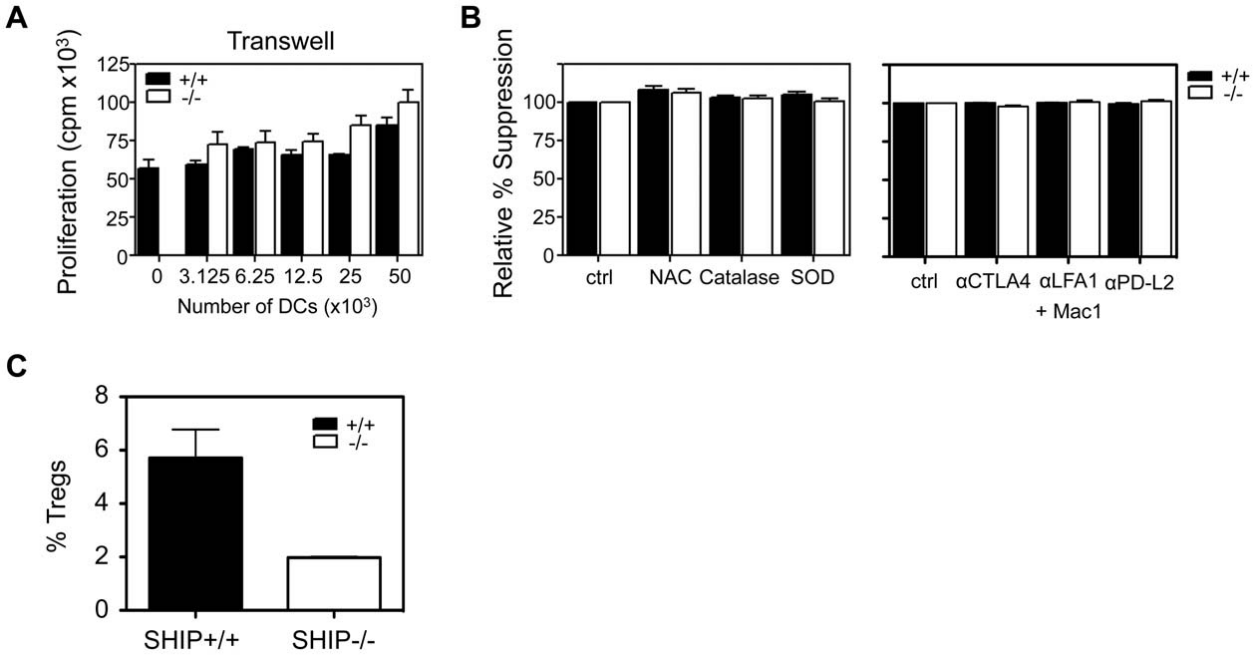
SHIP+/+ and −/− GM-CSF-derived DCs suppress via a contact-dependent mechanism. **A)** The indicated number of SHIP**+/+** and −/− GM-DCs were plated in the bottom chamber of a 0.4 µm 96 well transwell plate and WT splenocytes (2×10^5^ ) were stimulated with soluble αCD3+ αCD28 Abs and plated in the top chamber. Proliferation was determined after 72 hrs by incorporation of ^3^H-thymidine for the last 18 hrs. Data shown are the mean ± SEM of triplicate cultures and are representative of 3 independent experiments. **B)** Left panel, relative percent suppression with the addition of agents that reduce the presence of ROS (2 mM NAC, 100 U/ml catalase, 200 U/ml SOD). Right panel, relative percent suppression with the addition of blocking antibodies to CTLA4 (10 µg/ml), LFA1+ mac1 (5 µg/ml each) and PD-L2 (10 µg/ml). Data shown are the mean ± SEM of triplicate cultures and are representative of at least 2 independent experiments with the exception of PD-L2 which was only performed once. **C)** SHIP+/+ and −/− GM-DCs were cultured for 4 days with WT sorted conventional T cells at a ratio of 1∶2 DCs to T cells and analyzed for Treg induction by flow cytometry. Data shown are the mean ± SEM of two independent experiments.

T cell suppression can be mediated by soluble cytokines, by direct cell-cell contact, or both [35]. To determine if suppression was contact dependent, since it did not appear to be mediated by known immunosuppressive cytokines, we carried out transwell studies. As shown in Fig 5A, separation of αCD3+ αCD28-activated T cells from SHIP+/+ or −/− GM-DCs by a semi-permeable membrane abrogated suppression at all cell doses tested. This is in agreement with WT GM-DCs suppressing via an NO-dependent mechanism, since although not necessarily requiring direct cell contact, close proximity is required because of the short half-life (5 seconds) of NO. Reactive oxygen species (ROS) have also been implicated in phagocyte-induced T cell suppression [36], and like NO, require close proximity to exert their effects. To determine if ROS were involved in either SHIP+/+ or −/− GM-DC-induced T cell suppression, we added the ROS scavengers N-acetyl-cysteine (NAC), catalase or superoxide dismutase (SOD) to activated T cell cultures containing either SHIP+/+ or −/− GM-DCs. As shown in Fig 5B (left panel), addition of these ROS scavengers had no effect on T cell proliferation. These results demonstrate that although the mechanism of suppression requires close proximity, it is not ROS dependent.

To determine if specific contact molecules on the surface of SHIP+/+ or −/− GM-DCs were responsible for T cell suppression, we blocked the inhibitory receptor CTLA-4 or the adhesion molecules, LFA-1+ Mac-1 (CD11b), with neutralizing Abs. Under both conditions no change in the level of suppression was detected (Fig 5B, right panel). Also, since it was recently reported [37] that alternatively activated, M2 macrophages express programmed death ligand 2 (PD-L2) and that blockade of PD-L2 prevented M2-macrophage-induced suppression of T cells we asked if SHIP+/+ or −/− GM-DCs (which exhibit several hallmarks of M2 macrophages) were using PD-L2 to mediate their contact-dependent suppression of T cell proliferation. However, neither SHIP+/+ or SHIP−/− GM-DC-induced suppression was reversed with a blocking Ab to PD-L2 (Fig 5B, right panel).

We also tested if suppression could be occurring indirectly through the induction of Tregs. Treg induction can occur both through contact-dependent and -independent mechanisms [14], [38], [39]. However, SHIP−/− GM-DCs induced fewer Tregs when co-cultured with conventional WT T cells (ie, CD4^+^CD25^−^CD45RB^hi^) suggesting, at least, that this is likely not the mechanism of suppression employed by SHIP−/− GM-DCs (Fig 5C).

## Discussion

**Figure 6:**
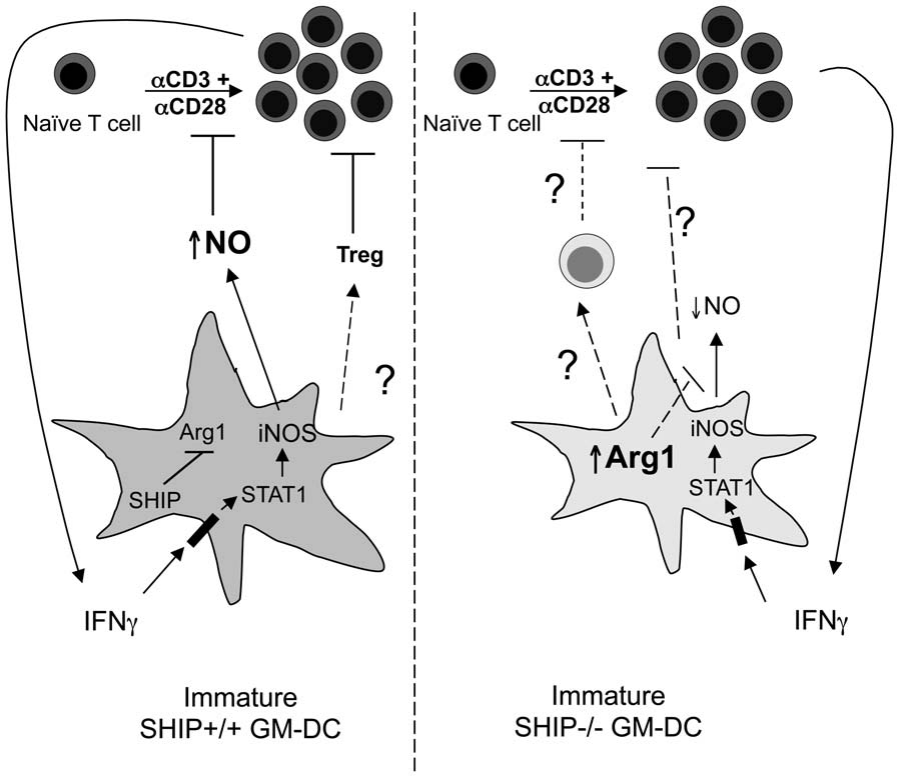
Model of SHIP+/+ and −/− GM-DC-induced T cell suppression. SHIP+/+ and −/− GM-DCs both suppress T cell proliferation in a contact-dependent manner. αCD3+ αCD28-stimulated T cells secrete IFNγ, which acts on WT GM-DCs to upregulate iNOS and secrete NO. This NO then suppresses T cell proliferation. SHIP−/− GM-DCs express Arg 1 and do not produce NO, but may use an alternate direct mechanism of suppression or induce the expansion or differentiation of a regulatory cell, likely not Tregs, to suppress T cell proliferation. If a second cell type is involved in SHIP−/− GM-DC-induced suppression, its induction or activation is contact-dependent.

In this study we compared the ability of SHIP+/+ and −/− splenic, Flt3L and GM-CSF-derived DCs to suppress polyclonal T cell proliferation and found that both SHIP+/+ and SHIP−/− GM-DCs have an intrinsic ability to suppress T cell proliferation while splenic and FL-DCs do not. Upon further investigation of the mechanism of suppression employed by these GM-DCs, we discovered that SHIP+/+ GM-DCs use, in part, a close proximity-dependent, IFNγ-induced NO production mechanism, possibly in concert with induced Tregs (see Model, Fig 6). SHIP−/− GM-DCs, on the other hand, do not produce significant amounts of NO, likely because of high Arg 1 expression, and their suppression of αCD3+ αCD28-induced T cell proliferation cannot be reversed through IFNγ neutralization or inhibition of iNOS.

A number of studies have been conducted to investigate the influence of DC culture conditions on the ability of the derived DCs to suppress T cell activation *in vitro*. Based on these studies, the dose of GM-CSF as well as the presence or absence of IL-4 used in culture was found to impact the resultant phenotype [40]. Specifically, DCs derived from BM with low GM-CSF concentrations were found to be phenotypically immature and induced T cell unresponsiveness. In addition, these cells were much more resistant to LPS, TNFα and CD40-induced maturation, but were sensitive to the effect of IL-4-induced maturation. On the other hand, DCs derived with high doses of GM-CSF were more mature and showed little phenotype/functional difference with the addition of IL-4 [40]. These studies [40], [41] did not elucidate a mechanism of action of T cell unresponsiveness beyond the suggestion that the immature phenotype prevented activation. In our current studies, DCs were cultured in the presence of high doses (10 ng/ml) of GM-CSF. Rossner *et al*, on the other hand, found that the non-DC fraction (CD11c^−^) of 8–10 day low GM-CSF cultures and 3–4 day high GM-CSF cultures suppressed T cell activation via a contact and NO-dependent mechanism [10]. This group classified these cells as *in vitro-*derived MDSCs. These results are similar to those we obtained with our SHIP+/+ CD11c^+^ day 8, high concentration GM-CSF cultures. Consistent with our results, it has been reported that rat BM-derived DCs, but not splenic DCs produce NO and are capable of T cell suppression [42]. As well, like us, Taieb et al found that rat Flt3-derived DCs could not suppress T cell proliferation [9]. Unfortunately, as already mentioned, many factors can influence the DCs generated, including not only GM-CSF concentration but the age of the mice used, starting cell density, mechanical stress and batch to batch variation in fetal calf serum [43]. This, together with the finding that DCs may differ somewhat if derived with recombinant GM-CSF versus GM-CSF from conditioned media, makes literature comparisons difficult. In addition, the way in which T cells are activated also appears to influence the ability of myeloid cells to suppress, at least in the case of MDSCs [22].

Intriguingly, our results demonstrate that DCs generated in the presence of GM-CSF from SHIP−/− BM are capable of suppression, but that this suppression is not reversible by any means tested, including those that reversed the suppressive activity of SHIP+/+ GM-DCs. Not all mechanisms of suppression are direct. A study using human DCs showed that regulatory DCs induce CD4^+^CD25^+^ Tregs, which are capable of suppressing T cell responses [44]. However, we found that SHIP−/− GM-DCs were less capable than WT GM-DCs at inducing Tregs, suggesting this indirect mechanism is likely not responsible for SHIP−/− GM-DC-induced T cell suppression. As well, given that one of the primary mechanisms of Treg-induced suppression is via TGFβ [14], we found that neutralizing TGFβ or adding LAP did not affect the level of suppression of either SHIP+/+ or SHIP−/− GM-DCs (Fig 4A), further suggesting Tregs are not likely a large component of the suppressive mechanism.

In conclusion, we show that, unlike FL-DCs and splenic isolated DCs, GM-CSF-derived WT and SHIP−/− DCs are capable of suppressing polyclonal T cell proliferation. It appears that WT GM-DCs suppress, at least in part, via a contact and IFNγ-dependent induction of NO while SHIP−/− DCs are incapable of NO production and express high levels of the enzyme Arg 1, yet are still equally suppressive, perhaps because of the immature phenotype of these DCs [21]. Thus far, only prevention of contact is able to reverse T cell suppression by SHIP−/− GM-DCs which suggests that they could be particularly good at preventing graft versus host disease or prolonging allograft survival in mice because of a reduced likelihood that they will be converted to immunogenic DCs *in vivo*. This finding could be applicable to a clinical setting through the use of either inhibitors of SHIP or the use of RNA interference to reduce SHIP levels in human BM-derived DCs prior to transplant. Further understanding of the unique mechanism of T cell suppression utilized by SHIP−/− DCs will likely reveal other targets for the pharmacological manipulation of DC suppressive functions.

## Materials and Methods

### Ethics Statement

This study was carried out in strict accordance with the recommendations set out by the Canadian Council on Animal Care. The protocol was approved by the University of British Columbia Animal Care Committee (protocol #A07-0503).

### Mice

SHIP+/+ and −/− mice, backcrossed onto a C57Bl/6 background for at least 12 generations (provided by Dr Frank Jirik, University of Calgary, Calgary, AB) were used between 6–12 weeks of age. Mice were maintained in the Animal Resource Centre of the British Columbia Cancer Research Centre under specific pathogen-free conditions.

### Generation of GM-CSF-derived DCs

Red blood cell lysed bone marrow (BM) cells were cultured in IMDM containing 10% FCS, 0.00125% (v/v) MTG, 2 mM glutamine, 100 U/ml penicillin/streptomycin and 10 ng/ml rmGM-CSF (GM-DCs). Cells were seeded at 6×10^5^ cells/well (1 ml) in 12 well plates and 1 ml of fresh cytokine containing medium was added on day 3. On days 5 and 7, half the cell-free supernatant was replaced with fresh cytokine containing medium. Non-adherent cells were harvested on day 8 and DCs enriched by EasySep® CD11c-PE positive selection (StemCell Technologies, Vancouver) according to the manufacturer's instructions.

### Generation of Flt3L-derived DCs

Red blood cell lysed BM cells were cultured at 1.5×10^6^ cells/ml in RPMI containing 10% FCS, 100 U/ml penicillin/streptomycin, 50 µM β-ME and 100 ng/ml rmFlt3L. Cells were seeded at 4.5×10^6^ cells/well (3 ml/well) in 6 well plates and left for 8 days after which non-adherent cells were harvested as Flt3L-derived DCs (FL-DCs) and used in subsequent experiments.

### Splenocyte preparation and splenic DC isolation

Spleens were harvested from WT mice and the cells extracted by resuspending and passing through a 100 µm cell strainer. Red blood cells were lysed with NH_4_Cl solution at a 1 volume cells: 3 volumes NH_4_Cl for 5–10 min on ice and the remaining cells washed and resuspended in IMDM containing 10% FCS, 0.00125% (v/v) MTG, and 100 U/ml penicillin/streptomycin. For splenic DC isolation, SHIP+/+ and −/− splenocytes were washed and the DC population enriched using EasySep® CD11c-PE positive selection (StemCell Technologies Inc.) according to the manufacturer's instructions.

### Nitric oxide assay

NO production was determined indirectly by measuring the accumulation of nitrite (NO_2_^−^), a stable breakdown product of NO, in the tissue culture supernatant using a modification of the Griess assay [45], [46]. Briefly, 50 µl of supernatant was sequentially incubated with equal volumes of 1% sulfanilamide in 2.5% phosphoric acid and 0.1% phenylnapthylenediamine dihydrochloride in 2.5% phosphoric acid at 23°C. After 5 min, the absorbance of samples at 570 nm was determined and NO_2_^−^ concentration calculated by comparison to a NaNO_2_ standard curve.

### T cell suppression assay

This assay was performed according to the protocol of Thornton and Shevach [47] with a few modifications. SHIP+/+ or −/− BMDCs or splenic DCs were plated at 5×10^4^ cells/well in a 96 well flat bottom plate and serial 1∶2 dilutions performed down to 3.125×10^3^ cells/well. Prepared splenocytes were stimulated with 0.5 µg/ml αCD3+2.5 µg/ml αCD28 (eBioscience, San Diego, CA) (to stimulate T cell proliferation) and added (2×10^5^ cells/well) alone or to the DC-containing wells in 200 µl total volume. Cells were incubated at 37°C for 72 hrs, with ^3^H-thymidine (2 Ci/mmole, 1 µCi/well) added for the last 18 hrs. The contents of each well were then harvested onto filtermats and counted using an LKB Betaplate Harvester and Liquid Scintilation Counter (LKB Wallac, Turku, Finland). Neutralizing Abs to IL-4 were from eBioscience (San Diego, CA), to IL-10 and CTLA-4 from BD Biosciences (Mississauga, ON, Canada), to IL-13, IFN-γ, IL-6 and TGF-β from R&D Systems (Minneapolis, MN). Catalase, N-acetyl-L-cysteine (NAC), superoxide dismutase (SOD), and non-specific NOS inhibitor N^G^-monomethyl-L-arginine (L-NMMA) were from Sigma-Aldrich (St. Louis, MO). Recombinant human latency-associated peptide (LAP) was from R&D Systems and carboxy-2-Phenyl-4,4,5,5-tetramethylimidazoline-1-oxyl-3-oxide (PTIO), an NO scavenger, was from Cayman Chemicals (Ann Arbor, MI). [(S)-(2-Boronoethyl)-L-cysteine] (BEC), a competitive inhibitor of Arg1 and 2 that does not inhibit iNOS, was generously donated by Dr J.-L.Boucher. (Universite Paris Descartes, Paris, France) and exiguamine A by Dr. Ray Anderson (Vancouver, BC). TIB-218, a rat IgG2aκ Ab selective for the β subunit of mouse LFA-1 and CD11b (CD18) (αLFA-1+ MAC1), was purified from hybridoma supernatants in house. Recombinant mouse IFN-γ and IL-2 were from StemCell Technologies (Vancouver, BC, Canada). When used, these were added to DC-containing wells just prior to the addition of the activated splenocytes. Percent suppression of proliferation was calculated as follows:
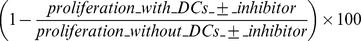
Relative percent suppression of proliferation was calculated as
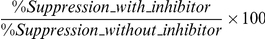



In parallel, similar assays were carried out in 48-well (600 µl total volume) plates to allow supernatant collection and analysis by ELISAs. Transwell experiments were conducted in 96 well 0.4 µm transwell plates (Corning, Lowell, MA) in 250 µl total volumes. DCs were plated in the bottom chamber and stimulated splenocytes in the top chamber. The 0.4 µm semi-permeable membranes that separate the upper and lower chambers allow diffusion of soluble materials but not cell migration. Control conditions, consisting of wells containing only activated splenocytes in the top chamber and media in the bottom chamber were also performed.

### RNA isolation and quantitative real-time PCR

RNA was purified from GM- or FL-DCs using TRIzol according to the manufacturer's instructions. Reverse transcription was used to generate cDNA and qPCR was performed using SYBR green. The primers used for qPCR analysis were the following: *β-actin* forward, ACTAATGGCAACGAGCGGTTC and reverse, GGATGCCACAGGATTCCATACC; *Arg 1* forward, CAGAAGAATGGAAGAGTCAG and reverse, CAGATATGCAGGGAGTCACC; *Arg 2* forward, ACAGGGTTGCTGTCAGCTCT and reverse, TGATCCAGACAGCCATTTCA; *Nos2* forward, CGAAACGCTTCACTTCCAA and reverse, TGAGCCTATATTGCTGTGGCT and *Indo* forward, AGAGCTCGCAGTAGGGAACAG and reverse, CATCACCATGGCGTATGTG. Reactions were carried out in an ABI 7900 real-time PCR machine (Applied Biosystems). Values are expressed relative to actin.

### Treg Induction

WT conventional T cells (CD4^+^CD25^−^CD45RB^hi^) were cultured (RPMI 1640 supplemented with 10% FBS, 10 mM HEPES, 2 mM glutamine, 1 mM sodium pyruvate, 1 mM MEM non-essential amino acid solution, and 100 U/ml each of penicillin G and streptomycin) in the presence of plate-bound αCD3 (10 µg/ml, 2C11) and co-stimulated with SHIP+/+ or −/− DCs (2∶1 ratio T cell to DC) in the presence of rhIL-2 (100 U/ml; Chiron). After 4 days, cells were harvested and analyzed by flow cytometry for Treg induction based on expression of CD4 (clone L3T4) and Foxp3 (clone FJK-16s) (eBioscience).

### Statistical analysis

Statistical significance was calculated using a two-tailed unpaired student *t* test using Microsoft excel or GraphPad Prism. Differences were considered significant when p<0.05.
